# Upregulation of miR-101 during Influenza A Virus Infection Abrogates Viral Life Cycle by Targeting mTOR Pathway

**DOI:** 10.3390/v12040444

**Published:** 2020-04-15

**Authors:** Shipra Sharma, Anirvan Chatterjee, Purnima Kumar, Sunil Lal, Kiran Kondabagil

**Affiliations:** 1 Department of Biosciences and Bioengineering, Indian Institute of Technology Bombay, Powai, Mumbai 400076, India; 2 Virology Group, International Centre for Genetic Engineering and Biotechnology, New Delhi 110067, India; 3 School of Science, Monash University, Bandar Sunway 47500, Malaysia

**Keywords:** Influenza A virus, mTOR, micro RNA, cellular pathway, virus replication

## Abstract

Micro RNAs (miRNAs) are a class of small non-coding single-stranded RNA, which play an important role in modulating host-Influenza A virus (IAV) crosstalk. The interplay between influenza and miRNA interaction is defined by a plethora of complex mechanisms, which are not fully understood yet. Here, we demonstrate that in IAV infected A549 cells, a synchronous increase was observed in the expression of mTOR up to 24 hpi and significant downregulation at 48 hpi. Additionally, NP of IAV interacts with mTOR and modulates the levels of mTOR mRNA and protein, thus regulating the translation of host cell. RNA sequencing and qPCR analysis of IAV-infected A549 cells and NP transfected cells revealed that miR-101 downregulates mTOR transcripts at later stages of infection. Ectopic expression of miR-101 mimic led to a decrease in expression of NP, a reduction in IAV titer and replication. Moreover, treatment of the cells with Everolimus, a potent inhibitor of mTOR, resulted in an increase of miR-101 transcript levels, which further suppressed the viral protein synthesis. Collectively, the data suggest a novel mechanism that IAV stimulates mTOR pathway at early stages of infection; however, at a later time-point, positive regulation of miR-101 restrains the mTOR expression, and hence, the viral propagation.

## 1. Introduction

Influenza A virus (IAV) is a group of RNA viruses of the Orthomyxoviridae family, endemic to wild aquatic birds, which serve as a natural reservoir; they are known to infect pigs, horses, ferrets, seals and several other mammalian species [1]. The genome of IAV is made up of eight single-stranded negative-sense RNA segments, with each segment forming a complex with ribonucleoproteins (RNPs) and viral RNA-dependent RNA polymerase (RdRp) consisting of three subunits, PB1, PB2 and PA [2,3]. As the IAV genome has limited coding capacity, its survival and propagation are intricately linked to its ability to challenge cellular anti-viral defenses and regulate cellular processes necessary for viral replication. IAV infection activates key host intrinsic signaling molecules, such as the PI3K/Akt [4] and MAP Kinase pathways [5,6], for efficient viral propagation and increasing the viral load on the host. Both pathways exploit the mechanistic target of rapamycin (mTOR) to promote protein synthesis on activating downstream effector proteins [7]. The mTOR protein is a 289-kDa serine/threonine kinase, a member of a PI3K-related kinase family that is known to be highly evolutionary conserved [8]. It acts as a critical nutritional and cellular regulator of protein synthesis, cell proliferation, motility and survival [9]. Furthermore, mTOR balances both autophagy and protein synthesis in response to cellular demands [10]. In order to establish its own replication, two IAV-encoded proteins known as NP and M2 induce autophagy through regulation of Akt/mTOR signaling pathway [11]. Besides being ubiquitously expressed in all eukaryotic cell types, mTOR is found to be upregulated in various cancers such as ovarian cancer [12], human hepatocellular carcinoma [13] and cutaneous melanoma [14]. A recent study demonstrated that IAV activates mTORC1 to likely promote translation to ensure viral gene expression and viral replication at later stages of the infection [15]. The centrality of this protein warranted a detailed study of the regulation of mTOR to explore new avenues for the prevention and treatment of “flu” pathologies.

miRNAs are a family of small noncoding RNAs that bind to the 3′ untranslated regions (3′ UTRs) of their target mRNA, leading to the inhibition of its transcription, thereby governing the repression of protein synthesis [16]. These small, conserved RNAs are involved in the regulation of several important biological processes, including cellular differentiation, proliferation, apoptosis and altered regulation of mRNA expression, and have been suggested to play a significant role in viral pathogenesis [17]. An established example is the modulation of mTOR activity in T-cells through post-transcriptional regulation of mTOR mRNA by the Let-7 miRNA [18]. mTOR is a validated target of miR-199a-3p and miR-100 miRNAs in hepatocellular carcinoma and ovarian cancer, respectively [19,20]. miRNA profiling studies have shown that they affect replication and pathogenesis of RNA viruses by directly binding to their genome or through virus-mediated changes in the host transcriptome. Interestingly, the replication of Hepatitis C Virus (HCV) was reduced when the liver-specific miR-122 binding to HCV RNA was blocked, indicating that miR-122 promoted HCV replication [21]. HIV-1 exploits the host miRNA cellular systems by upregulating Let-7c and miR-34a/miR-124a and downregulating their targets, p21 and TASK1, respectively, in order to enhance viral replication, pathogenesis and survival [22].

However, the role of miRNAs in the IAV-host network is still not fully understood. Previous studies have shown the expression of miRNAs across various subtypes of IAV and found that more than a hundred miRNAs were significantly altered in infected cells [23,24]. Among these, miR-4276 inhibits influenza infection by regulating the expression of cytochrome c oxidase subunit VIc, which in turn stimulates caspase-9 apoptotic pathway [25]. Non-structural-1A binding protein of the influenza virus is reported to be regulated by host-encoded miRNA-548an, which further facilitates viral infection [26].

In the present study, we aimed to profile miRNAs that are involved in Akt/mTOR pathway during IAV infection. First, we examined the activation of Akt and mTOR through a Ras family protein, N-Ras, in X-31 infected A549 cells. The levels of p-4EBP1 decreased by the inhibitory effects of mTOR, which in turn activated p-eIF4E, and hence, the synthesis of protein increased in the infected cells. Furthermore, we report mTOR as an interacting partner of NP that further modulated N-Ras/Akt/mTOR pathway and thus regulated protein synthesis of the host cell. Using RNA sequencing, we analyzed the differential regulation of miRNAs between X-31 infected A549 cells and NP transfected cells. miRNAs exhibiting significant deregulation included miR-101, which is known to repress mTOR levels by binding to the 3′-UTR of mTOR. Elevated levels of miR-101, both during IAV infection, and NP transfection, regulated mTOR expression and suppressed viral replication at later stages of infection. In concordance with these observations, it can be concluded that the specificity and dynamics of the Akt/mTOR signaling cascade during IAV infection are regulated by the temporal expression of miR-101.

## 2. Materials and Methods

### 2.1. Cells, Cell Lines, Plasmids and Viruses

A549 cells were obtained from American Type Tissue Culture (VA, USA) and maintained as per the supplier’s instructions. The NP gene of H5N1 A/Hatay/2004 isolate was cloned into the pCDNA 3.1-His plasmid [27]. The pHygEGFP construct containing a segment of the 3′-UTR of the mTOR mRNA (927 nt) containing predicted targets for miR-100 and miR-101 was provided by Dr Philip E. Pellett [28]. The X-31 virus, a reassortant between PR8 and A/Aichi/68(H3N2) IAV strain, was used at an MOI of 1 (unless specified) for 1 h at 37 °C. After 1 h absorption, cells were washed with PBS and were supplemented with DMEM and 0.3% bovine serum albumin (BSA) (Himedia, Mumbai, India) medium containing 1 μg/mL of tosylsulfonyl phenylalanyl chloromethyl ketone (TPCK)-treated trypsin (Sigma, St. Louis, MO, USA).

### 2.2. Antibodies miRNA Mimics and siRNA

p-mTOR, mTOR, p-S6K1, S6K1, N-Ras, p-Akt, p-4EBP1 and GAPDH antibodies were procured from Cell Signaling Technologies (Danvers, MA, USA). The NP antibody was obtained from Abcam (Cambridge, UK). miR-101 mimic, negative control mimic; mTOR siRNA and negative control siRNA were procured from Sigma-Aldrich.

### 2.3. Co-Immunoprecipitation (Co-IP)

Cells were harvested in the lysis buffer (50 mM Tris, pH 7.5, 150 mM NaCl, 1 mM EDTA and 0.1% Triton-X), and cell lysates were incubated with the primary antibody overnight at 4 °C followed by 90 min incubation with Protein A Sepharose beads (Genei Laboratories Pvt. Ltd., Bengaluru, India) at 4 °C. The beads were washed three times with chilled PBS, resuspended in 2X SDS dye and boiled, and eluates were resolved on SDS-PAGE [29].

### 2.4. Western Blotting

Cells were harvested in the lysis buffer supplemented with complete protease inhibitor cocktail (Roche, Basel, Switzerland). The lysates obtained were then subjected to SDS-polyacrylamide gel electrophoresis and transferred on PVDF membrane (Pall Corporation, New York, NY, USA) followed by probing with the indicated antibodies.

### 2.5. RNA Sequencing Analysis

To identify the miRNA involved in regulating the mTOR pathway, we performed small RNA sequencing of A549 cells infected with X-31 (MOI = 1) at 48 h post-infection and NP transfected A549 cells. Total RNA from cells was extracted using the RNeasy Mini Kit (Qiagen, Hilden, Germany)). Small RNA sequencing (SmRNA) libraries were prepared with TruSeq small RNA sample preparation protocol (Illumina, San Diego, CA, USA) at Genotypic Technology Pvt. Ltd., Bengaluru, India. Total RNA was used as starting material. Illumina Universal Adapter ligated fragments were reverse transcribed with Superscript III Reverse transcriptase (Invitrogen, Bengaluru, India). cDNA thus formed was enriched and barcoded by PCR amplification (15 cycles). An amplified library was size-selected in the range of 140–160 bp using Polyacrylamide gel by overnight gel elution and salt precipitation in presence of Glycogen (Invitrogen, Bengaluru, India), 3M Sodium Acetate (Sigma) and Absolute Ethanol (Tedia Company, Fairfield, CA, USA). Size selected pellet was re-suspended in nuclease-free water (Invitrogen, Bangalore). Illumina compatible sequencing library was initially quantified by Qubit fluorometer (Thermo Fisher Scientific, Waltham, MA, USA) and its fragment size distribution was analyzed on an Agilent 2200 TapeStation. Libraries were quantified by Kapa library quantification kit for Illumina (KAPA Biosystems, Wilmington, MA, USA). In this study, four small-RNA libraries (Uninfected (UI) vs. X-31 infected and Vector transfected (V) vs. Nucleoprotein transfected (NP)) were sequenced on an Illumina platform using single end 75 bp read chemistry. The srna-work benchV3.0_ALPHA1 [30] was used to trim 3’ adapter and perform length filtering (minimum length 16 bp and maximum 40 bp). All the sequences were aligned to the Homo sapiens genome (hg38) using bowtie-1.1.12 [31]. Aligned reads were extracted and checked for ncRNA (piRNA, rRNA, tRNA, snRNA and snoRNA) contamination. The unaligned reads to ncRNAs database were mapped to known miRNA as catalogued in miRbase-21 [32]. Repeated miRNA reads were de-duplicated by clustering approach using CD-HIT3 [33] and the read count profile was generated. Differential gene expression (DGE) analysis was outperformed using DESeq [34] tool implemented in the R ver 3.4 statistical package. Variations in the reads were normalized using the DESeq library.

### 2.6. Quantification of Transcripts by Real-Time Quantitative PCR 

Total RNA from cells was extracted using the RNeasy Mini Kit from Qiagen, and 500 ng of RNA was reverse-transcribed in a volume of 20 μL with the commercial cDNA kit (Genetix Biotech Asia Pvt. Ltd., Mumbai, India). Using SYBR green chemistry (Agilent Technologies, Santa Clara, CA, USA), 1 μL of cDNA was used in a qPCR reaction volume of 20 μL run on Stratagene Mx3000P (San Diego, CA, USA) real-time PCR instrument. The housekeeping gene GAPDH was used to normalize the Ct values obtained in the real-time PCR reactions, which were then used to calculate fold changes compared to the mock sample using the ΔΔCt method. The list of primers for all the probed miRNAs with sequences are in Table S1.

### 2.7. Stem Loop qPCR

The expression levels of mature miRNAs were analyzed using stem-loop end point PCR with minor modifications [35]. One µg total RNA was reverse-transcribed using 1 µL of Stem-loop RT Primer (100 ng/µL) in a volume of 20 μL reaction. The RT enzyme was inactivated by incubating the reaction at 85 °C for 5 min. One μL of direct cDNA was used for PCR using miRNA specific forward primer and universal reverse primer (5′-GTGCAGGGTCCGAGGT-3′) to get an amplification product. A 2% agarose gel was used to visualize the RT product. One μL of cDNA was used in a SYBR Green based qPCR reaction in a volume of 20 μL using a real-time PCR instrument, Stratagene Mx3000P. U6 small nuclear RNA was used as an endogenous control for evaluating expression profiles of miRNAs. A detailed list of primers with sequences is in Table S2.

### 2.8. Flow Cytometric Analysis

Cells were fixed in 2% paraformaldehyde (PFA), permeabilized with 0.1% Triton X-100, and then incubated with anti-NP antibody and Alexa Fluor 488–conjugated goat anti-rabbit secondary antibody. The cells were analyzed with a FACS Aria III flow cytometer (50000 cells per sample; BD Biosciences, USA), and the data were analyzed with FlowJo software [36].

### 2.9. Immunofluorescence Microscopy

A549 cells were transfected with either pcDNA3.1-myc/His (control transfection) or pcDNA-NP and pHygEGFP-mTOR plasmids using lipofectamine 2000 following the manufacturer’s protocol (Invitrogen, Carlsbad, CA, USA). Cells were fixed at 48 h post transfection with 2% paraformaldehyde in PBS for 20 min at room temperature, followed by permeabilization with 0.4% Triton X-100 for 15 min at room temperature. The nucleus was stained with DAPI. Slides were observed under ×60 magnification of confocal laser scanning microscope (Carl Zeiss, Oberkochen, Germany). A549 cells infected with X-31 influenza virus at an MOI of 2 or 5 were fixed at different time points in PBS with 2% paraformaldehyde for 20 min at room temperature, permeabilized with 0.4% Triton X-100 in PBS for 15 min at room temperature, and blocked with PBS containing 5% bovine serum albumin. Immunostaining was performed using mouse anti-NP and rabbit anti-mTOR antibodies. Unbound-antibodies were washed away with PBS and incubated with Goat anti-rabbit Alexa 568 and Goat anti-mouse Alexa 488 conjugated antibodies purchased from Invitrogen (NY, USA). The nucleus was stained with DAPI. Slides were observed under 40× or 60× magnifications confocal laser scanning microscope.

### 2.10. Cell Viability Assay

A549 cells were seeded at 10,000 cells/well in a 96-well dish. After adherence, they were treated with either DMSO or 10 nM Everolimus for 24 h. Following this, 200 µg/µl of MTT (Sigma Aldrich) solution/well was added and incubated at 37 °C for 30 min to allow for the formation of formazan. The medium was removed, and 200 μL DMSO was added to each well to dissolve the formazan. Absorbance was measured on an ELISA plate reader with a test wavelength of 570 nm and a reference wavelength of 630 nm to obtain a sample signal (A570–A630). DMSO was used as a reference.

### 2.11. Statistical Significance

Data are expressed as means ± s.e.m. The statistical significance of results was calculated using Student’s *t*-test. A *p*-value of <0.05 and < 0.01 was considered to be significant.

## 3. Results

### 3.1. Influenza Infection Activates the Cell Survival N-Ras/Akt/mTOR Pathway

**Figure 1:**
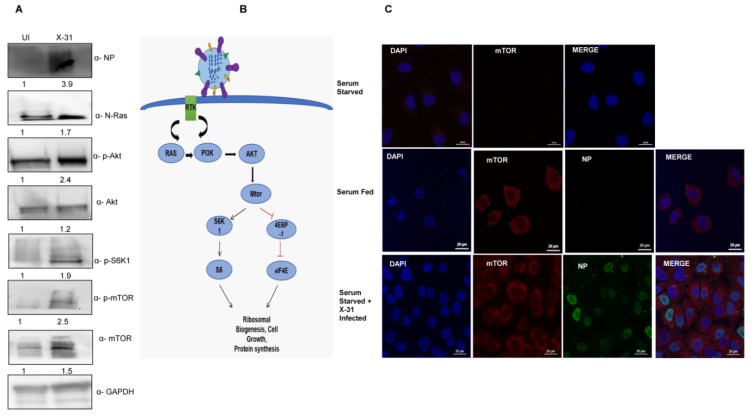
Induction of Akt/mTOR pathway by IAV. (**A**) X-31 infected A549 cells were infected at an MOI of 1 for 24 h or mock-infected (UI) in serum-starved cells and the whole-cell lysates from the same samples were resolved on SDS-PAGE for detection of NP, N-Ras, p-Akt, Akt, p-S6K1, p-mTOR, mTOR and GAPDH. (**B**) Schematic illustration for Ras/Akt/mTOR pathway. (**C**) Serum starved A549 cells (panel I) were uninfected, serum-fed cells were mock-infected (panel II) and serum-starved A549 cells infected with X-31 at MOI of 2 were fixed at 24 hpi and stained with DAPI for nucleus (blue), with goat anti-mouse antibody conjugated to Alexa 488 for NP (green), and with goat anti-rabbit antibody conjugated to Alexa-568 for mTOR (red). Scale is 20 µm.

As previously reported, IAV infection triggers the Akt pathway specifically at early stages of infection [5]. We further wanted to investigate whether IAV modulates the mTOR pathway, which is activated by the crucial signaling protein Akt, which in turn is activated by Ras proteins. To decipher the specific mechanism of stimulation of the mTOR pathway through N-Ras and Akt proteins, we infected the A549 cells with X-31 at an MOI of 1 for 24 h. Cell lysates, prepared 24 h after infection, were subjected to western blot analyses using antibodies specific for N-Ras, p-Akt, p-S6K1, p-mTOR and NP. As shown in Figure 1A, phosphorylation of Akt, S6K1 and mTOR was stimulated in X-31-infected cells relative to the uninfected cells. We examined the growth factors that activate mTOR through a signaling axis, PI3K/Akt and the downstream effectors of mTOR (Figure 1B).

A549 cells were mock-infected or infected with X-31strain of IAV at an MOI of 2 for 24 h. Cells were then either kept in standard growth medium or starved for all nutrients and growth factors. We found increased mTOR expression in serum-starved IAV infected A549 cells, compared to mock-infected, serum-starved cells (Figure 1C). As expected, mTOR was strongly expressed in the cytoplasm in uninfected, serum-fed A549 cells, while serum starvation completely abrogated the mTOR signal. This result suggests that the IAV activates the cell survival signaling pathway through N-Ras, Akt and mTOR.

### 3.2. Progressive Increase in mTOR Protein with Increase in Duration of IAV Infection

**Figure 2:**
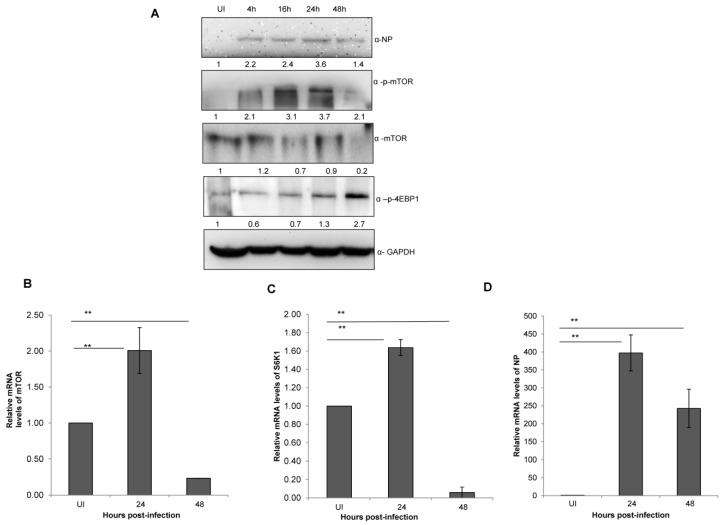
IAV infection stimulates an abrupt decrease in mTOR levels at later stages of infection. (**A**) A549 cells were infected with X-31 at an MOI of 1 and samples were collected at 0, 4, 16, 24 and 48 hpi. The whole-cell lysates from the samples were resolved on SDS-PAGE for detection of NP, p-mTOR, mTOR, p-4EBP1 and GAPDH. (**B**–**D**) A549 cells were either UI or infected with X-31 at an MOI of 1 and samples were collected at 24 and 48 hpi. Total RNA was isolated for the estimation of *mTOR* (B), *S6K1* (C) and *NP* (D) mRNA levels by qPCR using specific primers. The data in C, D and E are shown as mean ± S.D. of three independent experiments. * and ** indicate statistically significant differences at *p* < 0.05 and *p* < 0.01, respectively.

Next, we sought to determine whether X-31 virus infection alters the mTOR pathway protein expression in a time and virus dosage-dependent manner. A significant and progressive increase in the levels of p-mTOR, total mTOR and NP levels were observed upon infecting A549 cells with X-31 virus at increasing time of infection (Figure 2A) and MOI (Figure S1 in Supplementary Material). However, a considerable decrease was observed in p-mTOR and total mTOR protein levels, whereas p-4EBP1 levels increased moderately at 48 hpi, indicating that the former is regulating the levels of later causing the infected cells to decrease protein synthesis as the duration of infection increases. 

### 3.3. Influenza A Virus Positively Regulates mTOR Transcript Levels up to 24 h of Infection

Having assessed the effect of dose and time of IAV infection on mTOR signaling pathway, we next set out to investigate its impact on the process of mTOR transcription itself. Total RNA of X-31 infected A549 cells was extracted at 0, 24 and 48 hpi and quantified with qPCR. A two-fold increase was observed (*p* <0.01) until 24 hpi; however, an approximate 10-fold decrease of mTOR and S6K mRNA levels was observed at 48 hpi relative to 24 hpi (Figure 2B,C). Additionally, the NP transcript levels increased up to 24 hpi and decreased at 48 hpi (Figure 2D).

### 3.4. NP of IAV Interacts with mTOR and controls N-Ras-mTOR Pathway Proteins Expression 

**Figure 3:**
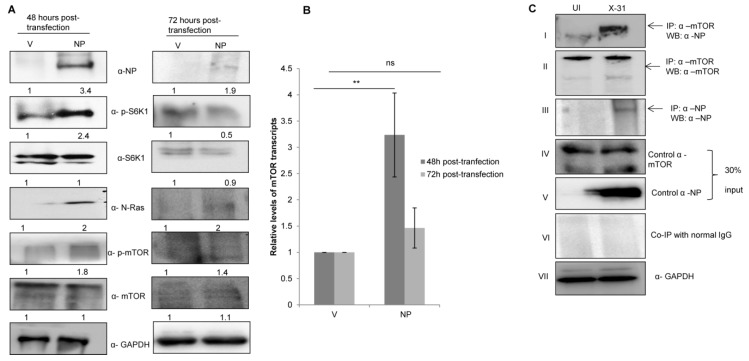
IAV NP interacts with mTOR in IAV-infected A549 cells and regulates the N-Ras/mTOR pathway. (**A**) A549 cells were transfected with either control (pcDNA3.1-myc/His) (V) or with His-NP (pcDNA3.1-myc/His-NP) (NP), 48 h and 72 h post-transfection and the whole-cell lysates were resolved on SDS-PAGE for the detection of NP, N-Ras, p-S6K1, total S6K1, p-mTOR, total mTOR and GAPDH using their respective antibodies. (**B**) A549 cells were transfected with V or with NP for 48 and 72 h. Total RNA was isolated for the assessment of *mTOR* transcripts by qPCR using specific primers. (**C**) X-31 infected A549 were harvested 24 hpi and prepared for co-immunoprecipitation assay. Panel I shows immunoprecipitation (IP) of mTOR by α-mTOR followed by western blotting (WB) with α-NP antibody. Panels II and III show immunoprecipitation followed by western blotting with α-mTOR and α-NP antibodies, respectively. Panel IV, V and VII show western blotting with α-mTOR, α-NP, and α-GAPDH antibodies. Panel VI shows the isotype control. ** indicate statistically significant differences at *p* < 0.01. ns denotes non-significant.

Given the role of NP in anti-viral host response and apoptosis at different stages of viral life cycle, we examined whether NP has a significant role in manipulating this pathway [37,38]. As shown in Figure 3A, phosphorylation levels of S6K1 and mTOR increased in cells transfected with NP as compared to the control cells. However, there was an appreciable decrease in the levels of NP, S6K1 and mTOR 72 h post transfection. Likewise, the *mTOR* mRNA expression increased 48 h post transfection; however, there was no significant change at 72 h (Figure 3B). The data indeed show that NP regulates the expression of N-Ras-mTOR pathway proteins until 48 h.

To determine whether NP and mTOR interacts during IAV infection, lung epithelial A549 cells were infected with the X-31 virus (MOI = 1). The infected lysates were subjected to immunoprecipitation using antibodies specific for NP and mTOR. NP of X-31 virus co-precipitated with mTOR protein (panel I). However, a pull down of NP with an α-mTOR antibody was inconclusive.

### 3.5. RNA Sequence Analysis of IAV Infected Cells and Identification of Differentially Expressed miRNAs

**Figure 4:**
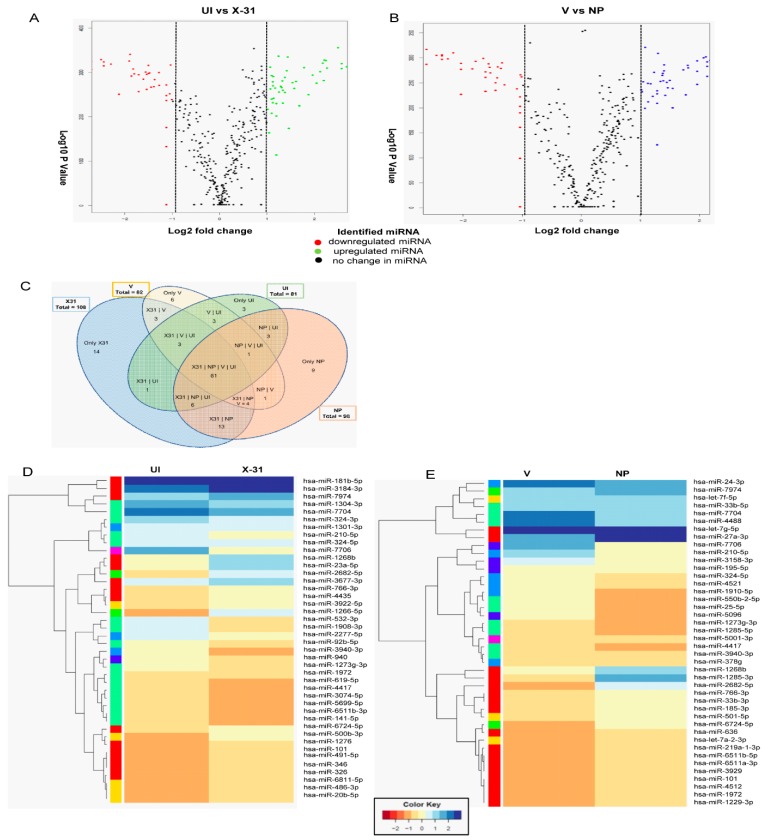
RNA sequencing identifies miRNAs involved in the regulation of the Akt/mTOR pathway. (**A**,**B**) The volcano plot represents the altered miRNAs in X-31 infected cells for 48 h at an MOI of 1 (SRR9688708 vs. SRR 9688709) and NP transfected 72 h sample (SRR9688707 vs. SRR9688706). miRNAs that were increased or decreased more than log2 FC (0.5) among the top 50 miRNAs are shown in green and red, respectively, whereas miRNAs with no significant change are shown in black. (**C**) This Venn diagram illustrates the numbers of miRNAs identified in each group and the number of common miRNAs within the four groups. (**D**,**E**) Heatmap showing the differential miRNA expression in A549 cells infected with X-31 for 48 h relative to mock-infected and NP transfected A549 cells for 72 h vs. the control cells were plotted using R package. miRNAs significantly changed (>two-fold) are colored in blue and red for upregulated and downregulated, respectively.

As shown in Figure 2C and D, IAV had a significant effect on *mTOR* transcript levels up to 24 h of infection. However, at 48 hpi, there is a substantial decrease in the levels of mTOR relative to the 24 hpi sample and control sample. The reduction in protein levels at a late stage of infection could be attributed to post-transcriptional regulation of *mTOR* by miRNA, which have been reported to be altered during influenza infection. Therefore, we investigated the possible role of miRNA in regulating the mTOR expression at post-transcription level to delineate the mechanism of the mTOR regulation at different stages of viral life cycle. To identify the miRNA involved in regulating mTOR pathway, we performed RNA sequencing and analysis of the dataset (SRR9688708, SRR 9688709, SRR9688707 and SRR9688706) in X-31 infected A549 (MOI = 1) at 48 hpi. The differential gene expression analyses of the above two data sets suggested a large number of miRNAs were upregulated or downregulated as validated by volcano plots (Figure 4A,B). Of the 105 miRNAs identified, which are involved in regulating the Akt/mTOR pathway, we observed that 19 miRNAs were positively regulated and 21 were negatively regulated in X-31-infected sample, relative to mock-infected, whereas 18 miRNAs were positively regulated and 22 were negatively regulated in NP transfected sample as compared to mock control, and no significant changes were observed in the rest. Interestingly, despite differences in experimental conditions, 61 miRNAs were found to be common in the four data sets (Figure 4C). Figure 4D,E show the heat maps of all four samples, which revealed the differences in expression level for these top 50 differentially expressed miRNAs, where features were clustered using a Euclidean distance metric. Of these positively regulated miRNAs, miR-101 was up-regulated in both the conditions, i.e., during IAV infection and in NP transfected cells.

### 3.6. Influenza A Virus Mediated Regulation of Cellular miRNAs

**Figure 5:**
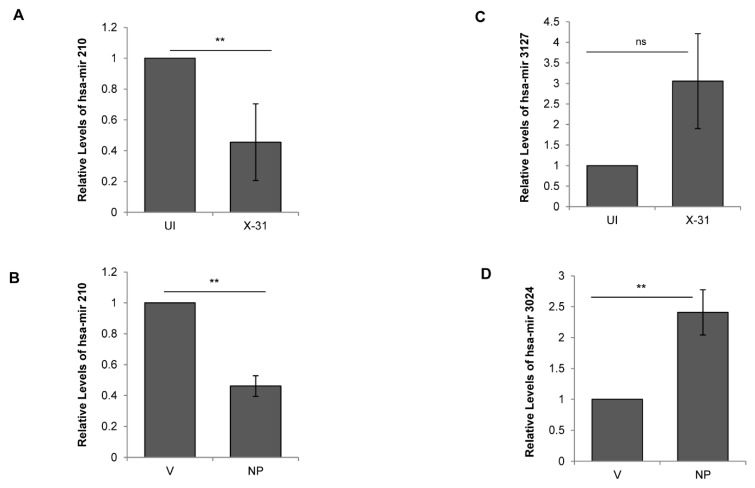
Regulation of cellular miRNAs during IAV infection. (**A**,**C**) A549 cells were infected with X-31 at an MOI of 1 and samples were collected at 48 hpi. Total RNA was isolated for quantitating miR-210 and miR-3127 transcript levels using stem-loop qPCR primers. (**B**,**D**) A549 cells were transfected with V or with NP for 72 h. Total RNA was isolated for the assessment of the level of miR-210 and miR-3024 transcripts by qPCR using specific primers. The data above is shown as mean ± S.D. of three independent experiments. ** indicate statistically significant differences at *p* < 0.01. ns denotes non-significant.

**Figure 6:**
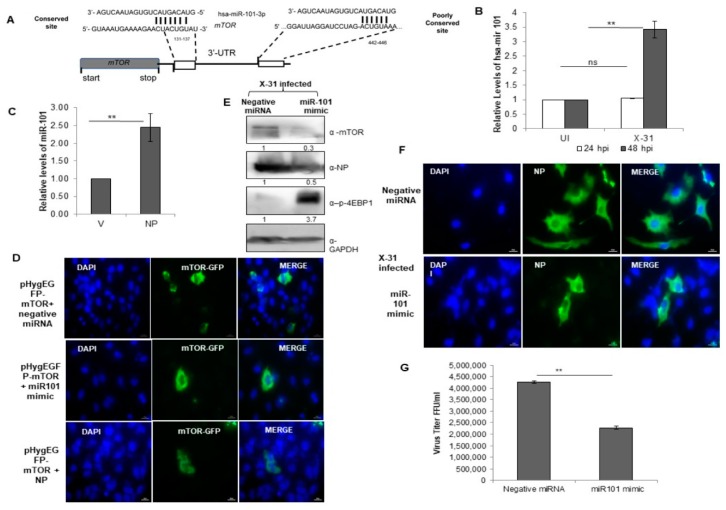
miR-101 is predicted to bind to 3′-UTR of the mTOR gene and upregulated during IAV infection. (**A**) Sequence complementarity between miR-101 seed region with the target site in the 3′UTR of mTOR. (**B**) A549 cells were infected with X-31 at an MOI of 1 and samples were harvested at 24 hpi and 48 hpi. Total RNA was isolated to estimate the miR-101 transcript levels using stem-loop qPCR specific primers. (**C**) A549 cells were transfected with V or with NP for 72 h. Total RNA was isolated for the assessment of the level of miR-101 transcripts by qPCR using specific stem loop primers. (**D**) A549 cells were transfected with 1µg pHygEGFP-mTOR that were co-transfected with 50 nM negative control miRNA, 50 nM miR-101 mimic and NP. Nuclei were stained with DAPI (Blue). Numbers of GFP fluorescent cells were counted to evaluate the difference between different experiments of triplicate determinations. Scale is 20 µm. (**E**) A549 cells were transfected with 50 nM negative control miRNA or 50 nM miR-101 mimic followed by infection with X-31 at an MOI of 1 for 48 h and the whole-cell lysates from the same samples were resolved on SDS-PAGE for detection of mTOR, NP, p-4EBP1 and GAPDH. (**F**) A549 cells were transfected with 50 nM negative control miRNA or 50 nM miR-101 mimic followed by infection with X-31 at an MOI of 5 for 24 h. The cells were fixed at 24 hpi. NP was detected using goat anti-mouse-antibody conjugated to FITC (green). Nuclei were stained with DAPI (Blue). The percentage of virus-infected cells as determined by NP-expression was enumerated. Scale is 20 µm. (**G**) A549 cells were transfected with 50 nM negative control miRNA or 50 nM miR-101 mimic followed by infection with X-31 at an MOI of 1 for 48 h followed by determination of viral titers by flow cytometry analysis with anti-NP antibody conjugated to Alexa Fluor 488. The data in B, C and G is shown as mean ± S.D. of three independent experiments. ** indicate statistically significant differences at *p* < 0.01. ns denotes non-significant.

To validate the results of the above miRNA expression analysis, we performed qPCR of five randomly selected miRNAs, viz; miR-181, miR-210, miR-101, miR-3127 and miR-3074, in all the four samples analyzed. We observed congruity in the expression patterns of miRNAs obtained by both the methods (RNA-seq and stem-loop qPCR). While miR-181 showed no significant change, miR-210 was downregulated in the X-31 infected cells and NP transfected cells (Figure 5A,B). Alternatively, miR-3127 was expressed and positively regulated only in X-31 sample, whereas miR-3074 was observed to be upregulated only in cells transfected with pcDNA3.1-myc/His-NP construct (Figure 5C,D). Interestingly, miR-101 was observed to be positively regulated by a notable fold change of 2.29 and 2.43 in both X-31 and NP samples, respectively, as compared to their controls (Figure 6B,C). Together, these results supported the conclusion that miR-101, miR-3127 and miR-3074 were upregulated, while miR-210 was downregulated during IAV infection of human A549 cells (Table S3). This analysis identified a number of miRNAs that potentially regulate genes significant to the mTOR pathway, from which we selected miR-101 for more detailed investigation, as it showed considerable changes both in IAV-infected cells and NP transfected cells in the stem loop qPCR data.

### 3.7. miR-101 Is Upregulated during IAV Infection at Later Stages of Infection

To get further insight into the target site of miR-101, we examined it in greater detail using the bioinformatics tools, Targetscan [39] and miR database. The predicted target site of miR101-3p in the 3′-UTR of mTOR mRNA is highly conserved at position 131–137 and this target site is well-conserved in several organisms (Figure 6A). Notably, the transcripts of miR-101 were upregulated approximately three-fold at 48 hpi in IAV infected cells, whereas there was no change in the relative levels of miR-101 at 24 hpi (Figure 6B). Likewise, a two-fold increase was observed in the levels of miR-101 in NP transfected cells (Figure 6C). Since a change in the levels of the miR-101 transcripts at 48 hpi were in concordance with the earlier observed change in the transcripts and protein levels of mTOR, this impelled us to understand the underlying mechanism of regulation of the Akt/mTOR pathway during the later stages of influenza infection. To this end, an immunofluorescence assay was performed, where we transfected a plasmid construct, pHygGFP-mTOR and negative miRNA in A549 cells and checked for the expression of GFP tagged mTOR. Next, we transfected the same construct with a mimic of miR-101 and observed a significant decrease in the expression of mTOR. On further transfecting GFP construct with pcDNA-NP plasmid, there was a marginal increase in the expression of mTOR as compared to the former (Figure 6D, Figure S3A), indicating that miR-101 indeed has a binding site in mTOR mRNA and NP of IAV positively regulates the mTOR protein, and hence, the cellular response during the IAV life cycle.

### 3.8. miR-101 Suppresses IAV Infection

We further sought to investigate the regulatory role of miR-101 during IAV replication in mammalian cells. A549 cells were treated with either negative miRNA or miR-101 mimic followed by infection with X-31 virus for 48 h. This resulted in the inhibition of expression of mTOR and NP, which further led to an increase in levels of phospho-4EBP1, as confirmed by western blot analyses (Figure 6E). To assess in more detail the role of miR-101 in IAV replication, we examined the infectivity of A549 cells on transfecting its mimic. Compared to 50% of cells infected in the control at 48 h respectively only ~16% of cells were infected in miR-101 mimic treated cells (Figure 6F, Figure S3B). Consistent with the previous results, the viral titer was significantly curtailed by two-folds in the miR-101 mimic treated infected cells relative to control as calculated by flow cytometry for NP positive cells (Figure 6G). Silencing of mTOR using specific siRNA inhibited viral protein synthesis (Figure S2A) and virus production (Figure S2B) by about one and a half fold, an effect similar to that of miR-101 and its mimic. Collectively, these results indicate that miR-101 plays a critical role in the replication of influenza virus by negatively regulating the mTOR expression, which in turn, modulates the replication and translation of viral proteins.

### 3.9. Everolimus Upregulates hsa-miR101 and Hence Inhibits IAV Infection

**Figure 7:**
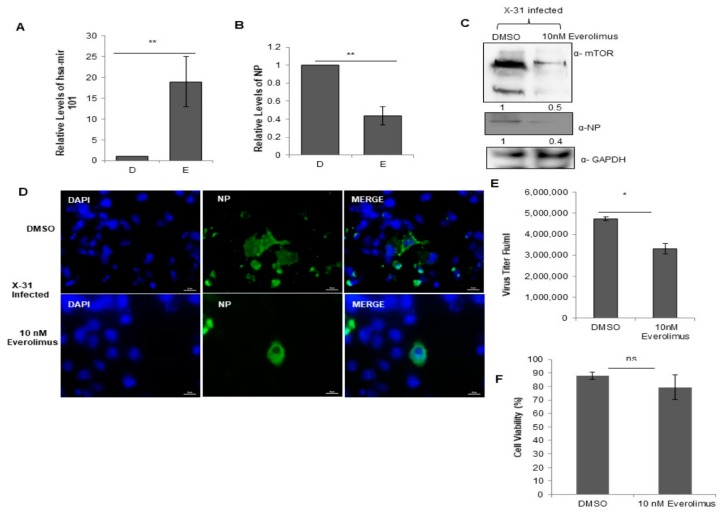
Treatment with Everolimus inhibits IAV infection. (**A**,**B**) A549 cells were treated with DMSO (0.1%, v/v) and 10 nM Everolimus for 12 h, followed by infection with X-31 at an MOI of 1. The samples were harvested at 24 hpi for extraction of RNA followed by quantification of miR-101 transcripts and NP transcripts. (**C**) A549 cells were treated with DMSO and 10 nM Everolimus for 12 h, followed by infection with X-31 at an MOI of 1. Samples were collected at 24 hpi and were resolved on SDS-PAGE for the detection of mTOR, NP and GAPDH. (**D**) A549 cells were treated with DMSO (0.1%, v/v) and 10 nM Everolimus for 12 h, followed by infection with X-31 at an MOI of 5. The cells were fixed 24 hpi. Nuclei were stained with DAPI (Blue). NP was detected using goat anti-mouse-conjugated to Alexa-488 (green). Scale is 20 µm. (**E**) A549 cells were treated with DMSO (0.1%, v/v) and 10 nM Everolimus for 12 h, followed by infection with X-31 at an MOI of 1 for 48 h followed by determination of viral titers by flow cytometry analysis with anti-NP antibody conjugated to Alexa Fluor 488. (**F**) A549 cells seeded in 96-well plate were treated with DMSO (0.1%, v/v) and 10 nM Everolimus. Cell viability was determined by MTT assay 24 h after treatment. The data in A, B, E and F is shown as mean ± S.D. of three independent experiments. * and ** indicate statistically significant differences at *p* <0.05 and *p* < 0.01, respectively. ns denotes non-significant.

In order to explore further the physiological relevance of the interaction between NP and mTOR, we examined the effects of treatment with Everolimus, a well-known inhibitor of mTOR on IAV replication. For this purpose, we treated the cells with DMSO or 10 nM Everolimus followed by infection with X-31 (MOI = 1). Intriguingly, we found that the miR-101 transcript levels were considerably increased in the drug-treated cells as compared to the DMSO control (Figure 7A), indicating that the miR-101 levels are further increased during the obstructed expression of mTOR. Additionally, the NP mRNA and protein levels were decreased by 2.3-fold in drug-treated IAV infected cells (Figure 7B,C). Complementing these observations, it was found in immunofluorescence assay that the infectivity of the cells also diminished approximately three-fold in the Everolimus treated cells (Figure 7D, Figure S3C). We observed a decrease in the virus titer by about one and a half-fold in the Everolimus treated infected cells relative to DMSO control as calculated by flow cytometry for NP positive cells (Figure 7E). Viability of the 10 nM Everolimus treated A549 cells was determined 48 h post-treatment by measuring the cytotoxicity in the drug versus the DMSO (10% v/v) control. The result in Figure 7F shows that Everolimus treated cells were nearly 80% viable as compared to DMSO control. Taken together, our findings unravel a novel and critical role of miR-101 in regulating mTOR pathway during different stages of IAV life cycle.

## 4. Discussion

Influenza virus, like other intracellular pathogens, exploits the host cell machinery for its efficient replication. As reported earlier, Ras–PI3K signaling axis acts as a host-oriented mechanism for viral internalization [40]. Moreover, a recent study has shown that mTORC1 activation supports viral protein expression and replication specifically at later stages of IAV infection when the cell is under significant stress [15]. In the present study, we have also shown that the IAV infection activates N-Ras, which further phosphorylates Akt and activates the downstream mTOR signaling proteins. This may help the virus to ensure efficient virus replication and to support viral RNA translation. A progressive increase in the protein and mRNA levels of mTOR was observed till 24 h of infection, whereas at 48 hpi, a considerable decrease both at the protein and the transcript levels of mTOR was observed, suggesting that the survival of the cell is compromised at later stages. We hypothesize that the anti-viral defense mechanism of the host cell is triggered as the cellular machinery is hijacked by the virus for its own replication. Thus, we further explored the mechanism of mTOR regulation specifically at later stages of infection, to investigate the role of critical cellular molecule influencing the inhibition of *mTOR* transcripts and protein abruptly, after a contemporaneous increase, when the viral replication has also increased. 

Considering that numerous studies have shown that an expansive range of cellular mechanisms, such as cell signaling, cell differentiation, apoptosis and chromosome maintenance are mediated by miRNAs [16,41], they have emerged as vital regulators of viral infection, replication and pathogenicity [42]. Similar to other viruses, influenza virus mutually interacts with host miRNAs, which further regulate viral replication and are deemed responsible for host tropism. Previous studies have also documented the differential expression of miRNA post-infection with various strains of IAVs, including swine H1N2 virus-infected pig lungs [43] and H1N1 virus-infected mouse lungs [44] and A549 cells [45]. miRNAs have been shown to play key roles in the regulation of IAV-induced inflammatory response by controlling the pro-inflammatory intracellular signaling pathways. miR-302a is known to inhibit IAV replication by controlling interferon regulatory factor-5 (IRF-5) expression and cytokine storm induction [46]. Upregulation of miR-4776 in Influenza Virus infected primary human bronchial epithelial cells suppress IκB β, which further promotes cell survival [47]. Interestingly, the differential expression of approximately 100 miRNAs in host cell during IAV life cycle, demonstrate their potential roles in antiviral host defense response [23,48]; therefore, it becomes imperative to further study the roles of small RNAs. These studies suggest that miRNAs are involved in the regulation of different stages in the viral life cycle via targeting viral RNAs or host factors.

For the miRNA species that had the most consistent and significant changes in expression following IAV infection and NP transfection, we examined the miRNAs involved in the mTOR pathway. miR-101 was found to have predicted targets on the mTOR pathway signaling proteins, which play an important role in regulating the translation of capped mRNAs, cell size, cell growth, cell cycle, cell survival and cytoskeletal organization. A combinatorial effect of miR-100 and miR-101 inhibited the production of infectious progeny of Human Cytomegalovirus by targeting the mTOR pathway components [28]. Specifically, miR-101-3p.2 has a predicted target on 3′-UTR of mTOR mRNA and miR-101-5p has been predicted to bind to the 3′-UTR of rictor. A key observation was that miR-101 was upregulated three-fold at 48 hpi and on transient expression of miR-101 mimic that mitigates the mTOR-EGFP expression in cells. Importantly, the miR-101 mimic suppressed the IAV replication resulting in the significant reduction of viral titers approximately 2.5 fold. This gives strong evidence that miRNAs influence virus production.

The RNA sequencing analysis revealed miR-101 was up-regulated on the ectopic expression of NP in A549 cells, which was further confirmed by qPCR. Given the pivotal role of NP in IAV replication [27], it may be hypothesized that miR-101 directly regulates NP transcripts, and thus the protein level or it indirectly disrupts NP and mTOR interaction via negative regulation of mTOR expression in the cells; nevertheless, additional investigations are needed to prove it. A similar investigation has shown that miR-485 directly binds to a conserved site of PB1 mRNA to regulate viral replication, in H5N1-infected HEK293T cells following treatment with miR-485 mimics [49]. Here, we have also shown for the first time that Everolimus upregulates miR-101, and hence, attenuates IAV infection. 

Considering the numerous molecular targets of miRNAs and the increasing evidence of an association with viral infections, it appears that miRNAs have great potential as biomarkers for the diagnosis and treatment of clinical diseases. Currently, miRNA-based antiviral strategies have been developed for HCV infections [50]; however, such strategies have not been successfully applied against IAV infections. Stable analogs of miR-101 and Everolimus could be used as potential anti-virals to treat various subtypes of IAV infections in humans and animals. Further studies will be required to investigate the regulatory network for regulation of miR-101, which increases our understanding of molecular mechanisms of viral infections. 

**Figure 8:**
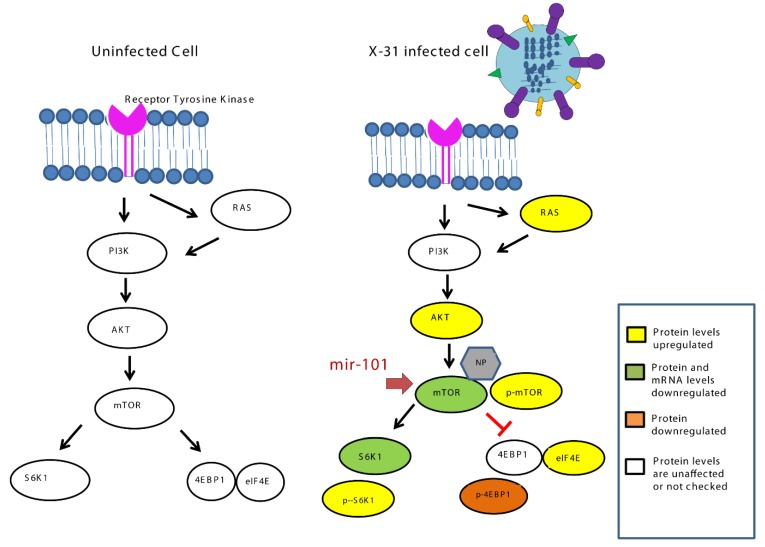
Schematic representation of mechanism for inhibition of IAV infection at later stage by upregulation of miR-101 which targets mTOR.

Our current findings highlight (Figure 8) how the miR-101/mTOR axis functions as a critical regulator of an IAV-induced manipulation of cellular machinery and provides a potential target for the therapeutic treatment of IAV infection in the future.

## Supplementary Materials

The following are available online at https://www.mdpi.com/1999-4915/12/4/444/s1, Figure S1: Reduction in mTOR levels at high MOI, Figure S2: Silencing mTOR has inhibitory effect on influenza virus A infection in A549 cells Figure S3: Quantitative Analysis of Immunofluorescence data Table S1: qPCR Primers Table S2: Stem loop qPCR primers Table S3: Expression of miRNA
