# Supplementary material for: Upregulation of miR-101 during Influenza A Virus Infection Abrogates Viral Life Cycle by Targeting mTOR Pathway
Source: Viruses. 2020 Apr 15;12(4):444. doi: 10.3390/v12040444 (PMC7232138; doi:10.3390/v12040444)
Supplement: Supplementary file 1 [file viruses-12-00444-s001.pdf]

SUPPLEMENTARY TABLE 1: qPCR Primers

	PRIMER	SEQUENCE
1	S6K1- FP	CAC ATA ACC TGT GGT CTG TTG CTG
2	S6K1-RP	AGA TGC AAA GCG AAC TTG GGA TA
3	mTOR-FP	CTG GGA CTC AAA TGT GTG CAG TTC
4	mTOR-RP	GAA CAA TAG GGT GAA TGA TCC GGG
5	GAPDH-FP	TCA CTG CCA CCC AGA AGA CTG
6	GAPDH-RP	GGA TGA CCT TGC CCA CAG C
7	NP-FP	CAGTACTGGGC ATAAGAC
8	NP-RP	GCATTGTCTCCGAAGAAATAAG

SUPPLEMENTARY TABLE 2: Stem loop qPCR primers

	PRIMER	SEQUENCE
1	hsa-miR-101	RT Primer-GTCGTATCCAGTGCAGGGTCCGAGGTATTCGCACTGGATACGACAGCATC<br><br>FP- GCGGCGGCAGTTATCACAGTGC
2	hsa-miR-210	RT Primer-GTCGTATCCAGTGCAGGGTCCGAGGTATTCGCACTGGATACGACCAGTGT<br><br>FP- GTAATTGAGCCCCTGCCCACCG
3	hsa-miR-3074	RT Primer-GTCGTATCCAGTGCAGGGTCCGAGGTATTCGCACTGGATACGACCTGGCT<br><br>FP- GCCACGGGTTCCTGCTGAACTG
4	hsa-miR-181	RT Primer-GTCGTATCCAGTGCAGGGTCCGAGGTATTCGCACTGGATACGACACCCAC<br><br>FP- GGCGCCGGAACATTCATTGCTGT
5	hsa-miR-3127	RT Primer-GTCGTATCCAGTGCAGGGTCCGAGGTATTCGCACTGGATACGACCTTCCC<br><br>FP- GCAGCGGATCAGGGCTTGTGGA
6	U6	RT Primer-PrGTCGTATCCAGTGCAGGGTCCGAGGTATTCGCACTGGATACGACAAAATATGGAAC<br><br>FP- TGCGGGTGCTCGCTTCGGCAGC<br>RP- CCAGTGCAGGGTCCGAGGT

SUPPLEMENTARY TABLE 3: Expression of miRNA

	Fold change	UI vs X-31	OV vs NP
Hsa-miR-101		2.29	2.43
Hsa-miR-210		0.3	0.16
Hsa-miR-3127		2.1	No expression
Hsa-miR-3074		No expression	2.5
Hsa-miR-181		No significant change	No significant change

Figure S1

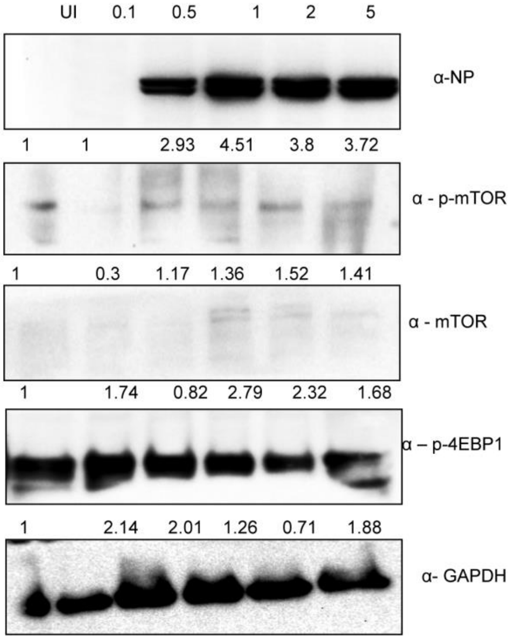

Figure S1. Reduction in mTOR levels at high MOI. A549 cells were either mock-infected (UI) or infected with X-31 at an MOI of 0.1, 0.5, 1, 2 and 5 for 24 h. The whole-cell lysates from the samples were resolved on SDS-PAGE for detection of NP, p-mTOR, mTOR, p-4EBP1 and GAPDH.

Figure S2

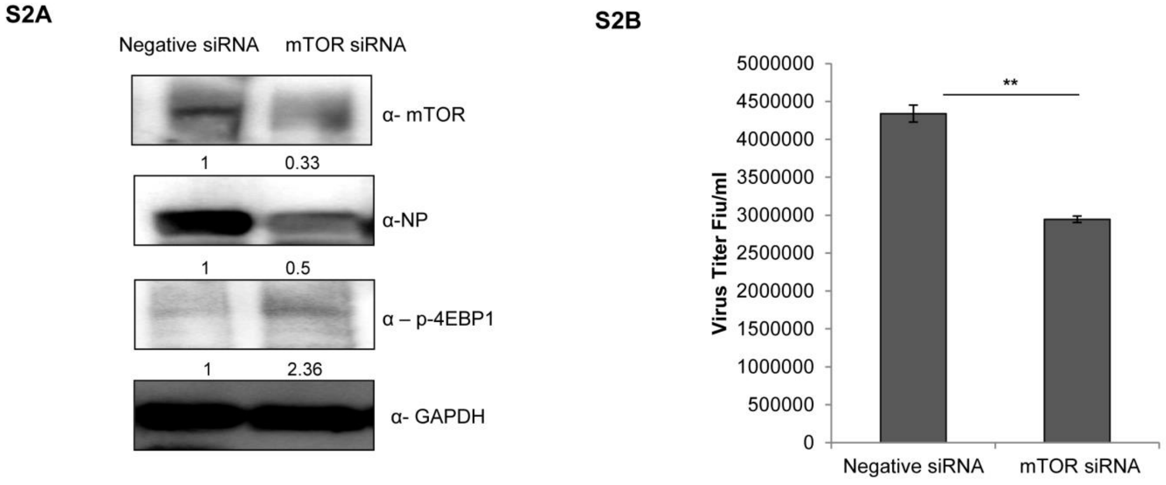

Figure S2. Silencing mTOR has inhibitory effect on influenza virus A infection in A549 cells. (A) A549 cells were transfected with either non-targeting (NT) or mTOR siRNA followed by infection with X-31 at an MOI of 1 and the whole-cell lysates from the same samples were resolved on SDS-PAGE for detection of mTOR, NP, p-4EBP1 and GAPDH. (B) A549 cells were transfected with 100 nM negative control siRNA or 100 nM mTOR siRNA mimic followed by infection with X-31 at an MOI of 1 for 48 h followed by determination of viral titers by flow cytometry analysis with anti-NP antibody conjugated to Alexa Fluor 488. The data in B is shown as mean± S.D. of three independent experiments. * and ** indicate statistically significant differences at $P < 0.05$ and $P < 0.01$, respectively.

Figure S3

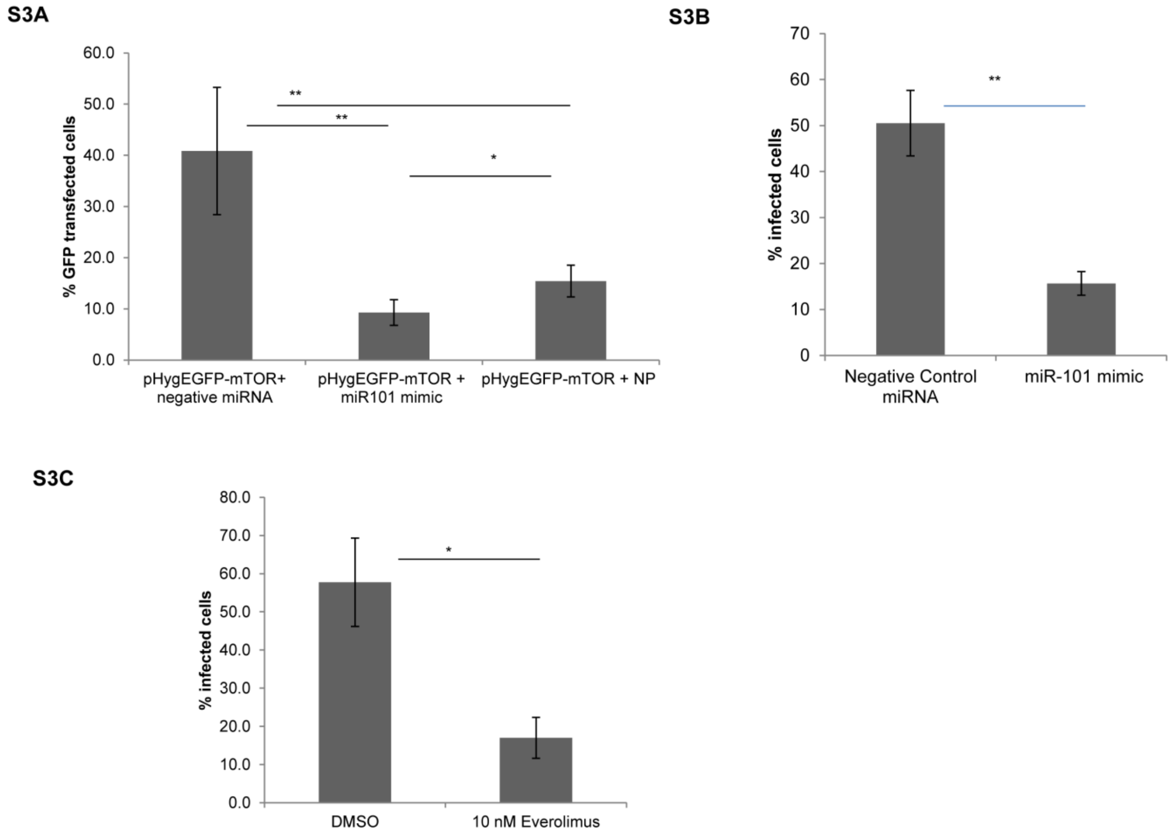

Figure S3. Quantitative Analysis of Immunofluorescence data. (A) Percentage of GFP transfected cells was calculated for 100 cells in 10 frames for pHygEGFP-mTOR that were co-transfected with 50 nM negative control miRNA, 50 nM miR-101 mimic and NP. (B) Percentage of IAV infected A549 cells was calculated for 100 cells in 10 frames and plotted for 50 nM negative control miRNA, 50 nM miR-101 mimic treated cells. (C) Percentage of IAV infected A549 cells was calculated for 100 cells in 10 frames and plotted for DMSO and 10 nM Everolimus treated cells.
